# Development and Validation of an Integrated Metabolism‐Obesity Index for Screening Renal Impairment in Patients With Newly Diagnosed Type 2 Diabetes Mellitus

**DOI:** 10.1155/jdr/5164386

**Published:** 2026-04-13

**Authors:** Xiangying Zhu, Xinyuan Li, Zhenhua Wang, Meixia Ji, Xiaoxiao Ji, Lihong Jin, Chunlai Zeng, Yuanliang Gu

**Affiliations:** ^1^ Department of Endocrinology, Lishui Hospital of Wenzhou Medical University, The First Affiliated Hospital of Lishui University, Lishui People′s Hospital, Lishui, Zhejiang Province, China; ^2^ Postgraduate Training Base Alliance of Wenzhou Medical University, Wenzhou, Zhejiang Province, China; ^3^ Center of Health Management, Lishui Hospital of Wenzhou Medical University, The First Affiliated Hospital of Lishui University, Lishui People′s Hospital, Lishui, Zhejiang Province, China; ^4^ Department of Public Health, Lishui Hospital of Wenzhou Medical University, The First Affiliated Hospital of Lishui University, Lishui People′s Hospital, Lishui, Zhejiang Province, China

**Keywords:** disease screening, metabolism, obesity, renal impairment, Type 2 diabetes

## Abstract

**Background:**

Diabetic kidney disease, a severe diabetes microvascular complication affecting 30%~40% of diabetic patients, raises end‐stage renal disease and mortality risks. This study is aimed at developing and validate a novel index combining obesity measures and metabolic parameters to screen renal impairment in newly diagnosed T2DM patients.

**Methods:**

A total of 387 and 259 newly diagnosed T2DM patients were recruited for model development and validation, respectively. Renal impairment was defined as eGFR < 60 mL/min/1.73 m^2^ and/or urinary albumin‐to‐creatinine ratio (UACR) ≥ 30 mg/g. Obesity‐ or metabolism‐related indicators were collected. Significantly independent indicators were selected via bidirectional stepwise regression in the modeling patients to develop a comprehensive index termed InMOI. In the validation patients, restricted cubic spline analysis examined the dose‐response relationship between InMOI and renal impairment, whereas ROC curves assessed the screening performance.

**Results:**

The triglyceride‐glucose index (TyG) combined with various obesity measures showed significant associations with renal impairment. The InMOI, derived from TyG‐BMI and TyG‐waist to hip ratio (TyG‐WHR), demonstrated a linear dose‐response relationship with the odds ratio for renal impairment. InMOI achieved an AUC of 0.78 for identifying renal impairment. Optimizing the index by replacing WHR with the visceral‐to‐subcutaneous fat area ratio measured by DEXA, the modified InMOI (m‐InMOI) showed a better performance (AUC = 0.81).

**Conclusion:**

This study underscores the value of metabolism‐obesity indices in early detection of diabetic renal damage and highlights the pathogenic role of visceral obesity and metabolic dysregulation in diabetic kidney disease. The InMOI provides a practical tool for large‐scale screening and early prevention of diabetic kidney disease.

## 1. Introduction

Diabetes mellitus represents a major global public health challenge, affecting approximately 500 million people worldwide, the majority of whom have Type 2 diabetes mellitus (T2DM) [1]. Affecting 30%–40% of individuals with diabetes, diabetic kidney disease stands as one of its most severe complications, markedly elevating the risk of end‐stage renal disease and all‐cause mortality [2]. Diabetic kidney disease, a primary manifestation of diabetic microvascular complications, involves complex pathological mechanisms, including metabolic disturbances, hemodynamic changes, and inflammatory responses [3].

Traditionally, the development and progression of diabetic kidney disease have been attributed to poorly controlled chronic hyperglycemia and hypertension. However, growing evidence indicates that obesity plays a critical role in the pathogenesis of diabetic kidney disease [4]. Obesity not only closely associates with T2DM but also exacerbates the risk of chronic kidney disease. Specifically, obesity aggravates renal impairment through multiple mechanisms: (1) inducing dyslipidemia and dysglycemia, leading to abnormal renal lipid deposition [2]; (2) sustaining glomerular hyperfiltration, which increases urinary albumin (UA) excretion rates [5]; and (3) promoting the secretion of pro‐inflammatory cytokines, accelerating renal fibrosis [6].

Notably, patients with both obesity and diabetes exhibit more severe insulin resistance and greater difficulty in glycemic control compared with those with diabetes alone [7]. Since insulin resistance is a central driver of diabetic kidney disease, accurate clinical assessment is essential, yet it remains challenging. Consequently, the triglyceride‐glucose index (TyG) has gained recognition as a cost‐effective surrogate for insulin resistance, replacing the need for the complex hyperinsulinemic‐euglycemic clamp [8]. Moreover, the TyG is more than just a metabolic marker; it is an independent contributor to renal microvascular damage [9–11]. Specifically, elevated TyG levels are associated with glomerular hyperfiltration, podocyte injury, and ectopic lipid deposition in the kidney [9–11]. Although body mass index (BMI) is a conventional metric for assessing adiposity, the BMI component itself possesses inherent limitations in predicting clinical outcomes. Specifically, BMI fails to distinguish between heterogeneous fat distribution patterns [12], leading to divergent renal risks among individuals with identical BMI scores. Empirical evidence of this limitation is underscored by the “obesity paradox [13]” and findings from the Look AHEAD trial [14]; the latter demonstrated that improvements in renal outcomes are primarily driven by the reduction of central adiposity (visceral fat) rather than total body weight. In light of these findings, it is imperative to move beyond simple anthropometric measures and adopt more integrated metabolic indices—such as those combining lipid profiles with adiposity—to capture the synergistic effects of obesity and metabolic dysfunction on renal health.

Recent research focuses have shifted from overall weight measurement to fat distribution characteristics, particularly visceral adipose accumulation, as a more accurate indicator of health risks [15, 16]. Central obesity (typically indicated by waist circumference [WC]) demonstrates a stronger association with renal impairment than general obesity. A study of overweight or obese T2DM patients revealed that reductions in fat mass and WC were significantly associated with improved renal outcomes, whereas changes in BMI showed no such correlation, underscoring the importance of assessing body composition beyond weight alone [17]. Although previous studies have investigated the relationship between baseline body composition and chronic kidney disease risk in T2DM patients, findings remain inconsistent. Longitudinal assessment of body composition changes may provide more comprehensive insights into chronic kidney disease risk than single baseline measurements [17, 18]. Furthermore, developing an integrated scoring system combining obesity metrics and metabolic parameters holds clinical significance for early identification of high‐risk patients.

This study is aimed at systematically analyzing the association between various obesity‐related indicators and renal impairment to develop a novel integrated index (InMOI) for predicting renal impairment risk in early‐stage T2DM patients. We focus on composite indicators derived from triglycerides (TGs), fasting glucose (FBG), and anthropometric measurements (e.g., waist‐hip ratio [WHR]) and validate the index using visceral‐to‐subcutaneous fat area ratio (VSR) measured by dual‐energy x‐ray absorptiometry (DEXA). The goal is to provide a practical and precise tool for early screening of diabetic kidney disease.

## 2. Methods

### 2.1. Study Design and Participants

This retrospective study utilized real‐world data from the Lishui People′s Hospital branch of the National Metabolic Management Center (http://national-mmc.com/). The study protocol was approved by the Ethics Committee of Lishui People′s Hospital (Approval No. 2025‐03‐22). As an observational study without additional interventions, all participants provided informed consent prior to enrollment. The study focused on patients with newly diagnosed T2DM to investigate the risk of renal impairment in the early stages of the disease. A total of 646 T2DM patients aged 18 to 40 years with a disease duration of ≤ 1 year were included. Patients with nondiabetic kidney disease, pregnancy, malignancy, severe hepatic dysfunction, or other conditions that might significantly affect metabolism were excluded. A total of 387 participants with measurements taken between January 2018 and December 2019 comprised the exploratory cohort for index development, whereas 259 participants with measurements between January 2020 and December 2021 constituted the validation cohort for index performance evaluation.

### 2.2. Indicator Calculation and Measurements

A comprehensive set of clinical indicators related to obesity and metabolism was systematically collected to capture risk features associated with renal impairment: (1) Anthropometric measurements—all measurements were taken by trained professionals using standardized instruments. WC was measured at the midpoint between the lower rib margin and the iliac crest. Hip circumference (HC) was measured at the fullest part of the buttocks. The WHR, waist‐to‐height ratio (WHtR), and BMI were calculated from the basic measurements; (2) biochemical measurements—fasting venous blood samples and first‐morning midstream urine samples were collected. Key indicators, including TG, FBG, glycated hemoglobin (HbA1c), serum creatinine (Scr), serum albumin (ALB), UA, and urinary creatinine (UCR), were analyzed using an automated biochemical analyzer; (3) composite indices—several composite indices, validated as proxies for insulin resistance and metabolic dysregulation, were calculated as candidate variables for the integrated index (Table S1); precise visceral fat measurement—the visceral fat area (VFA) and subcutaneous fat area (SFA) were quantified using DEXA, and their ratio (VSR) was calculated to validate and optimize the anthropometry‐based index.

### 2.3. Definition of Renal Impairment

Renal impairment was defined according to the Kidney Disease: Improving Global Outcomes (KDIGO) criteria as an estimated glomerular filtration rate (eGFR) of < 60 mL/min/1.73m^2^, calculated using the Chronic Kidney Disease Epidemiology Collaboration equation, or a UACR ≥ 30 mg/g.

### 2.4. Statistical Analysis

The statistical analysis followed a standardized pipeline from variable screening to model validation, performed using R software (Version 4.4.3): (1) Preliminary screening and univariate analysis—univariate logistic regression analyses were performed for each of variables, with the presence or absence of renal impairment as the dependent variable, adjusting for covariates such as age, gender, smoking, alcohol consumption, and diagnosed with history of hypertension. Concurrently, the Wilcoxon rank‐sum test was used to compare the distribution of each variable between T2DM patients with and without renal impairment. Variables that were statistically significant (*p* < 0.05) in both analyses were included in the subsequent multivariate analysis. (2) Multivariate analysis and index system construction—bidirectional stepwise regression (based on the Akaike Information Criterion, AIC) was applied to the candidate indicators to eliminate collinearity and identify core predictors independently associated with renal impairment. The regression coefficients (*β* values) from the final model were used as weights to construct the integrated metabolism‐obesity index. (3) Model performance validation and association assessment—in the independent validation participants, restricted cubic splines (RCS) were used to analyze the dose‐response relationship of integrated metabolism‐obesity index with the odds ratio (OR) for renal impairment. The area under the curve (AUC) of the receiver operating characteristic curve (ROC) was calculated to evaluate the discriminatory ability of the integrated metabolism‐obesity index for renal impairment.

## 3. Results

### 3.1. Study Population Characteristics and Inter‐Indicator Correlations

A total of 646 eligible patients with early‐stage T2DM were included in this study, with 387 subjects assigned to the derivation population and 259 to the validation population. In the derivation population, no significant differences were observed in baseline characteristics such as age, gender, or history of hypertension between the population with and without renal impairment (Table 1). Spearman correlation analysis among the obesity‐ and metabolism‐related indicators (Figure 1a) revealed significant correlations between certain indicators (e.g., WC and HC, TyG index and FBG), indicating potential multicollinearity in multi–indicator‐combined analyses.

**Table 1:** Demographic and clinical characteristics of participants.

Variables	Distribution in modeling patients	Distribution in validation patients	*p* value
Gender (*N*)			0.655
Male	296	202	
Female	91	57	
Age	34.51 ± 4.88	34.53 ± 4.68	0.850
Hypertension (*N*)			0.819
Yes	73	47	
No	314	212	
Smoking (*N*)			0.117
Yes	145	113	
No	242	146	
Alcohol use (*N*)			0.991
Yes	175	117	
No	212	142	
Renal impairment (*N*)			0.234
Yes	137	80	
No	250	179	
UACR	19.16 (9.52, 47.98)	19.35 (9.57, 39.76)	0.768
EGFR	95.79 (65.53, 135.86)	99.08 (59.06, 139.26)	0.560
BMI	26.69 ± 4.25	26.97 ± 4.94	0.456
BRI	4.34 ± 1.28	4.42 ± 1.63	0.498
GNRI	113.82 ± 11.30	114.27 ± 11.65	0.628
WWI	10.60 ± 0.65	10.59 ± 0.73	0.915
ABSI	0.08 ± 0.01	0.08 ± 0.01	0.874
WHtR	0.55 ± 0.06	0.55 ± 0.07	0.644
WHR	0.94 ± 0.06	0.94 ± 0.07	0.649
RFM	29.78 ± 6.40	29.64 ± 6.86	0.787
VSR	0.53 ± 0.18	0.50 ± 0.19	0.089
TyG	9.81 ± 0.89	9.91 ± 0.94	0.164
TyG‐WC	9.03 ± 1.48	9.19 ± 1.65	0.207
TyG‐BMI	262.41 ± 51.30	268.15 ± 58.62	0.200
TyG‐BRI	42.73 ± 13.51	44.00 ± 16.95	0.311
TyG‐GNRI	1118.01 ± 162.42	1134.55 ± 170.16	0.218
TyG‐WWI	103.96 ± 11.47	104.97 ± 12.26	0.291
TyG‐ABSI	0.78 ± 0.09	0.79 ± 0.09	0.288
TyG‐WHtR	5.37 ± 0.81	5.45 ± 0.92	0.245
TyG‐WHR	9.25 ± 1.15	9.37 ± 1.25	0.207
TyG‐RFM	292.00 ± 66.78	294.03 ± 73.90	0.722
TyG‐VSR	5.17 ± 1.90	4.97 ± 1.96	0.198


Abbreviations: ABSI, a body shape index; ALB, serum albumin; BMI, body mass index; BRI, body roundness index; FBG, fasting plasma glucose; GNRI, geriatric nutritional risk index; HC, hip circumference; RFM, relative fat mass; Scr, serum creatinine; TG, triglycerides; TyG, triglyceride‐glucose index; VSR, visceral‐to‐subcutaneous fat area ratio; WC, waist circumference; WHR, waist‐to‐hip ratio; WHtR, waist‐to‐height ratio; WWI, weight‐adjusted‐waist index.

Figure 1Identification and validation of the InMOI associated with renal impairment. (a) Spearman correlation heat map of multiple obesity/metabolic‐related variables in modeling participants (*n* = 387). (b) Bubble plot showing the association (−log_10_ *p* value from univariable logistic regression, *x*‐axis) and intergroup difference (−log_10_ *p* value from Wilcoxon test, *y*‐axis) of each variable with renal impairment. (c) Heat map of multicollinearity diagnostics (condition index and variance inflation factor) for models containing different combinations of significant indicators. The model incorporating TyG‐BMI and TyG‐WHR exhibited the least multicollinearity. (d) RCS analysis showing the positive linear association between the synthesized InMOI and the OR for renal impairment. (e) RCS curve showing the dose‐response relationship between InMOI and eGFR value. (f) Forest plot of the multivariable logistic regression analysis assessing the association of the InMOI with renal impairment after adjusting for covariates.

**Figure:**
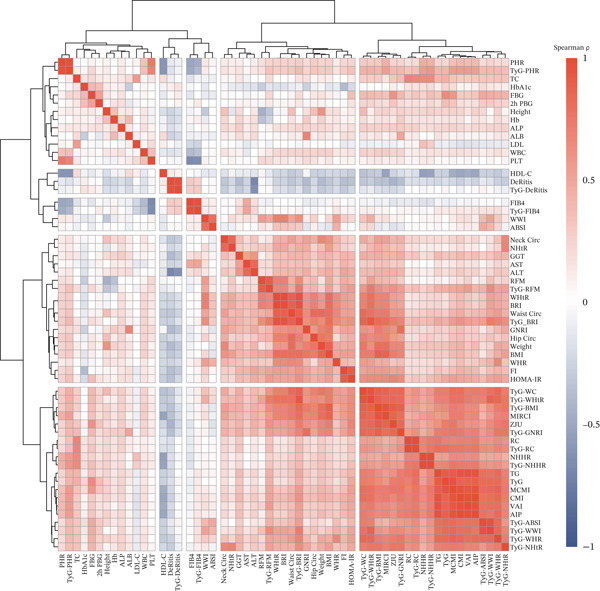
(a)

**Figure:**
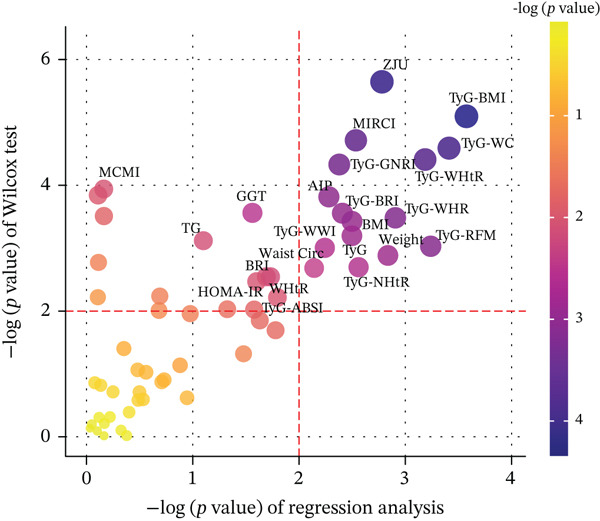
(b)

**Figure:**
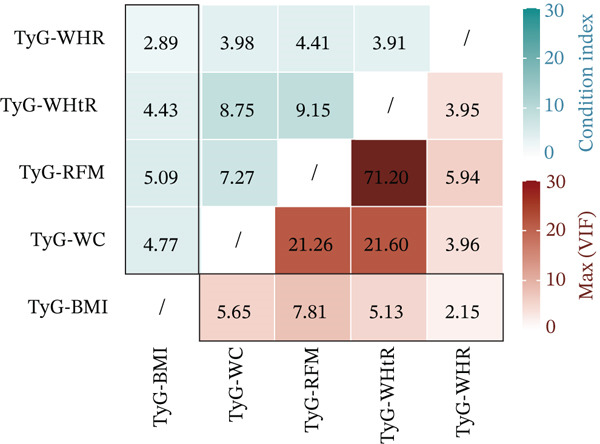
(c)

**Figure:**
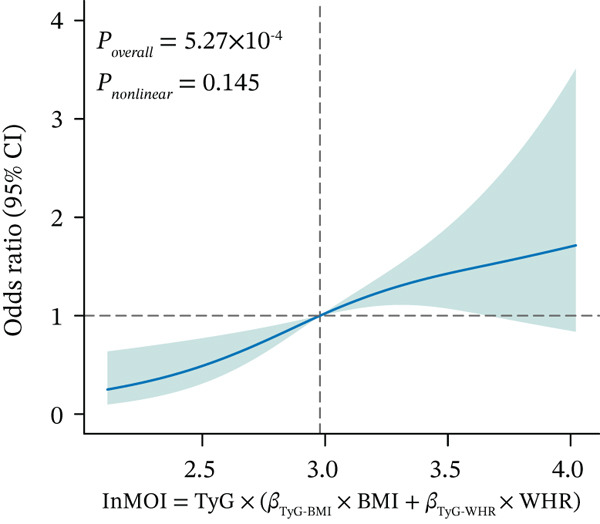
(d)

**Figure:**
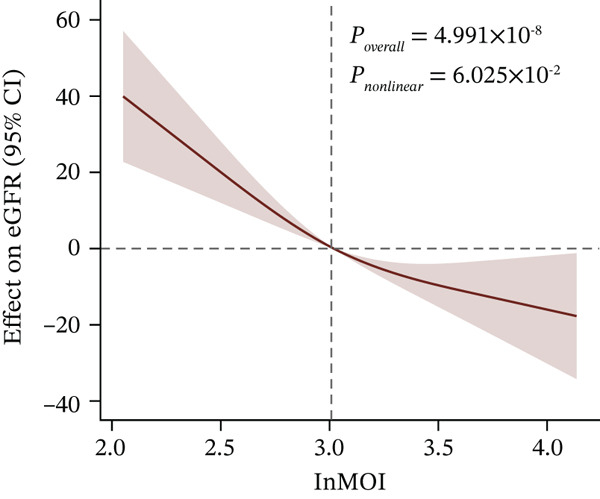
(e)

**Figure:**
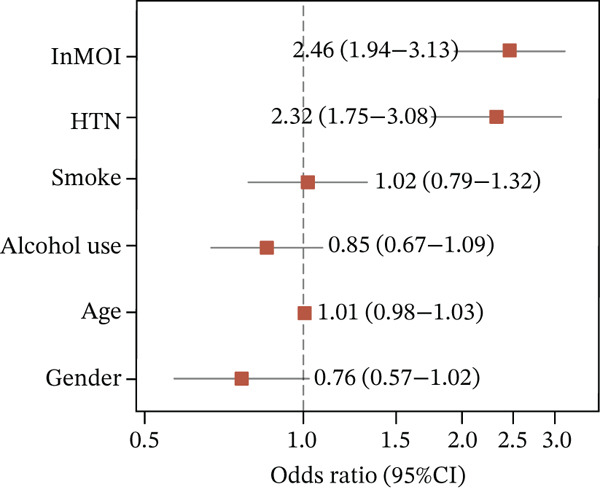
(f)

### 3.2. Preliminary Screening of Indicators Associated With Renal Impairment

Univariate logistic regression (adjusted for age, gender, smoking, alcohol consumption and history of hypertension) and Wilcoxon rank‐sum tests were used to identify indicators significantly associated with renal impairment. Figure 1b demonstrated that TyG index combined with various obesity measures (including TyG‐BMI, TyG‐WC, TyG‐WHtR, TyG‐WHR, etc.) was significantly associated with renal impairment. These indicators were included in subsequent multivariate analysis.

### 3.3. Multivariable Model Construction and Development of the InMOI

Using bidirectional stepwise regression (based on AIC), independent predictors were selected from the candidate indicators. The results indicated that TyG‐BMI and TyG‐WHR were independently associated with renal impairment (Figure 1c). Further assessment of multicollinearity in multivariable models showed that the model containing TyG‐BMI and TyG‐WHR (with covariates including age, gender, smoking, alcohol consumption and history of hypertension) had the weakest multicollinearity (all variance inflation factors [*V* *I* *F* *s*] < 2.0). The regression coefficients were used as weights to integrate TyG‐BMI and TyG‐WHR into a composite index, termed the InMOI. The formula is

InMOI=TyG×βTyG−BMI×BMI+βTyG−WHR×WHR

With the weights substituted, the simplified formula (details in Note S1) is

InMOI=TyG125×BMI+12.2×WHR

To further explore the dose‐response relationship between the InMOI and renal impairment, RCS analysis was performed. As shown in Figure 1d, a linearly increasing trend was observed between the InMOI and the OR for renal impairment (*P*_overall_ = 5.27 × 10^−4^; *P*_nonlinear_ = 0.145). Notably, the OR surpassed the threshold of 1.0 once the InMOI exceeded 2.95. Furthermore, the RCS analysis demonstrated a significant clinical correlation between the InMOI and eGFR as a continuous variable (*P*_overall_ = 4.99 × 10^−8^, *P*_nonlinear_ = 0.06), exhibiting a clear dose‐response effect (Figure 1e), whereby higher InMOI values were consistently associated with a progressive decline in eGFR. Specifically, the estimated effect on eGFR transitioned to a significant negative value once the InMOI exceeded 3.02, with the upper bound of the 95% CI falling below the zero line, further reinforcing the adverse impact of elevated InMOI on renal function. Figure 1f from multivariable logistic regression further confirmed that the InMOI (OR = 1.82,95*%*CI : 1.34 ~ 2.47) and history of hypertension (OR = 2.15,95*%*CI : 1.24 ~ 3.72) were independent predictors of renal impairment, whereas age, gender, smoking, and alcohol consumption showed no significant associations. To further elucidate the independent clinical significance of the InMOI beyond the potential confounding impact of HTN, we conducted comprehensive subgroup analyses stratified by HTN status (Figure S1 and Table S2). A consistent positive association between InMOI and renal impairment was observed in both HTN and non‐HTN participants across the modeling, validation and combined cohorts. (all P_overall_ < 0.05), as further evidenced by RCS analysis. Specifically, a consistent linear dose‐response relationship was observed regardless of HTN status (all P_nonlinear_ > 0.05), with the risk of renal impairment increasing progressively as InMOI rose.

### 3.4. Validation of the InMOI Performance in the Validation Cohort

In the validation population, individuals with renal impairment had significantly higher levels of TyG‐BMI, TyG‐WHR, and the InMOI compared with whom with nonimpaired (Wilcoxon test, *p* < 0.001) (Figure 2a). RCS analysis revealed a significant clinical correlation between the InMOI and eGFR as a continuous variable (P_overall_ = 8.14 × 10^−4^, P_nonlinear_ = 0.36; Figure 2b), illustrating a clear dose‐response pattern where higher InMOI levels were associated with a progressive decline in eGFR. A linearly increasing relationship with the OR for renal impairment (P_overall_ = 2.10 × 10^−7^, P_nonlinear_ = 0.59; Figure 2c), at which point the 95% CI remained entirely above 1.0 once InMOI surpassed 3.13. When the InMOI was divided into quintiles, the ORs for renal impairment showed a significant increasing trend (*p* for trend < 0.001) (Figure 2d). ROC analysis demonstrated that the InMOI diagnosed renal impairment with an AUC of 0.78 (95% CI: 0.72~0.84) (Figure 2e).

Figure 2Validation of the association between the InMOI and renal impairment in the validation participants. (a) Violin plots comparing the levels of TyG‐BMI, TyG‐WHR, and the InMOI between populations with and without renal impairment. All indicators were significantly higher in the T2DM patients with renal impairment. (b) RCS curve showing the nonlinear positive association between the InMOI and eGFR value. (c) RCS curve demonstrating a linear positive association between the InMOI and the OR for renal impairment. (d) Trend analysis of ORs for renal impairment across quintiles of the InMOI, showing an increasing trend. (e) ROC curve evaluating the diagnostic efficacy of the InMOI for renal impairment, with an area under the curve (AUC) of 0.78.

**Figure:**
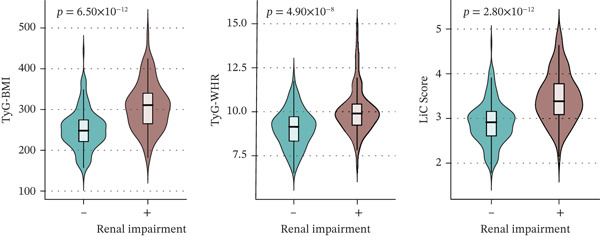
(a)

**Figure:**
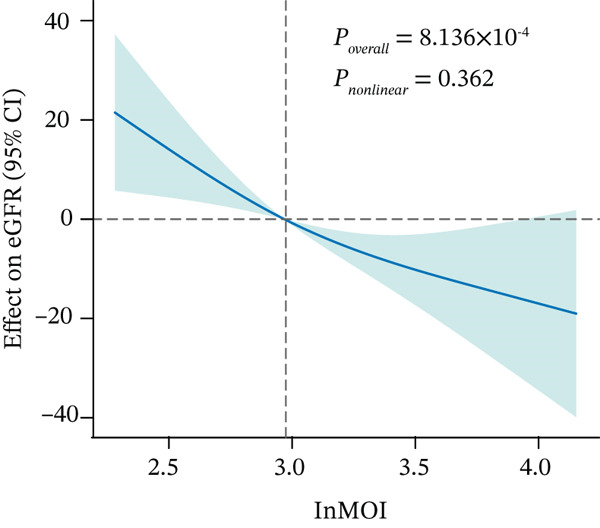
(b)

**Figure:**
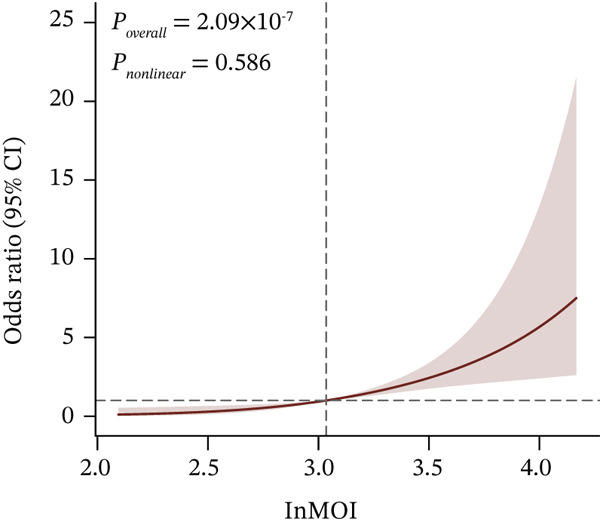
(c)

**Figure:**
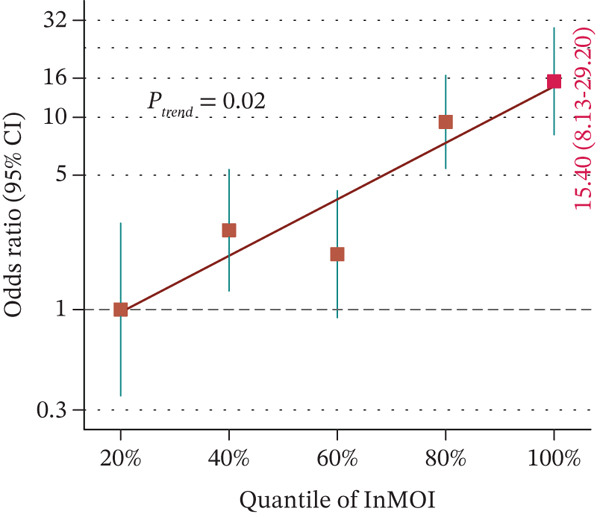
(d)

**Figure:**
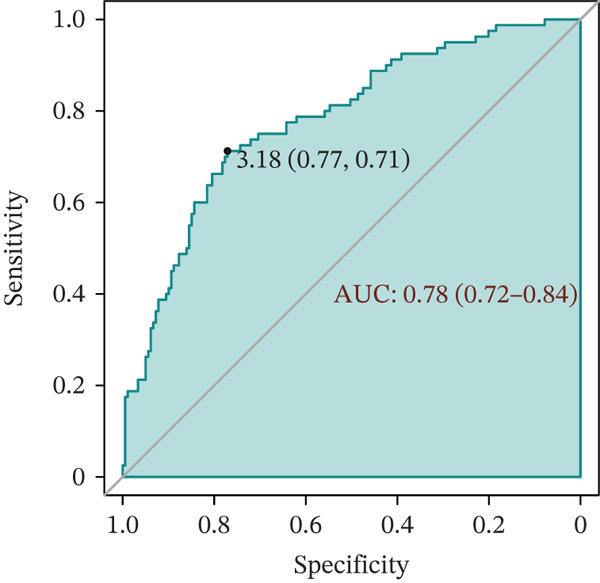
(e)

### 3.5. Index Optimization Based on DEXA‐Measured Fat Distribution (m‐InMOI)

To improve assessment precision, the VSR measured by DEXA was used to replace WHR, constructing a TyG‐VSR indicator. Multicollinearity tests indicated no significant collinearity among TyG‐VSR, TyG‐BMI, and the covariates (Figure S2). In the validation population, TyG‐VSR was significantly higher in patients with renal impairment (Figure S3) and showed a significant correlation with TyG‐WHR (Spearman *ρ* = 0.346, *p* < 0.0001) (Figure 3a). An optimized index, designated the m‐InMOI, was constructed using TyG‐BMI and TyG‐VSR. The formula is

m‐InMOI=TyG×βTyG−BMI×BMI+βTyG−VSR×VSR

Figure 3Development and validation of the m‐InMOI incorporating the TyG‐VSR indicator. (a) Scatter plot illustrating a significant positive correlation between TyG‐WHR and the more precise fat distribution metric TyG‐VSR. (b) Forest plot from multivariable logistic regression identifying the novel m‐InMOI (derived from TyG‐BMI and TyG‐VSR) and history of hypertension as independent factors significantly associated with renal impairment. (c) RCS curve showing a linear dose‐response relationship between the m‐InMOI and the OR for renal impairment in the validation population. (d) RCS curve showing the dose‐response relationship between m‐InMOI and eGFR value. (e) Trend of increasing ORs for renal impairment across ascending quintiles of the m‐InMOI. (f) ROC curve of the m‐InMOI for discriminating renal impairment in the validation population, achieving an AUC of 0.81.

**Figure:**
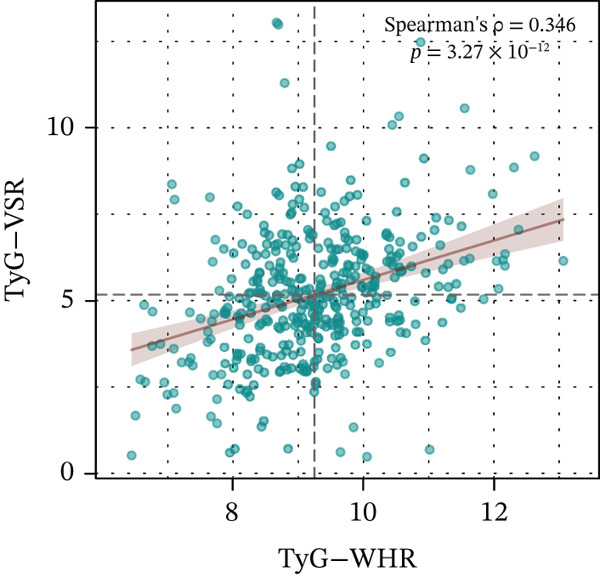
(a)

**Figure:**
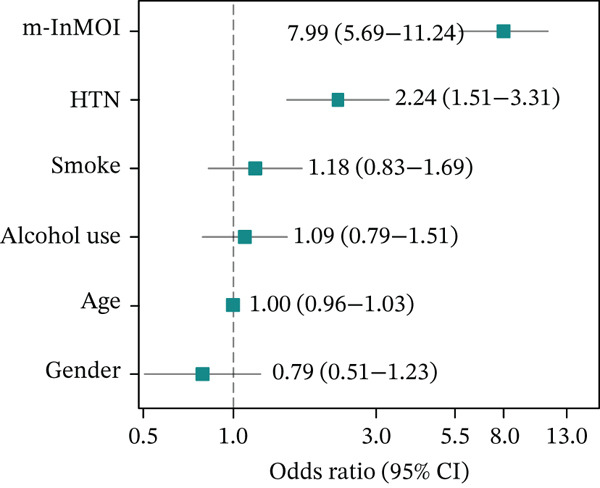
(b)

**Figure:**
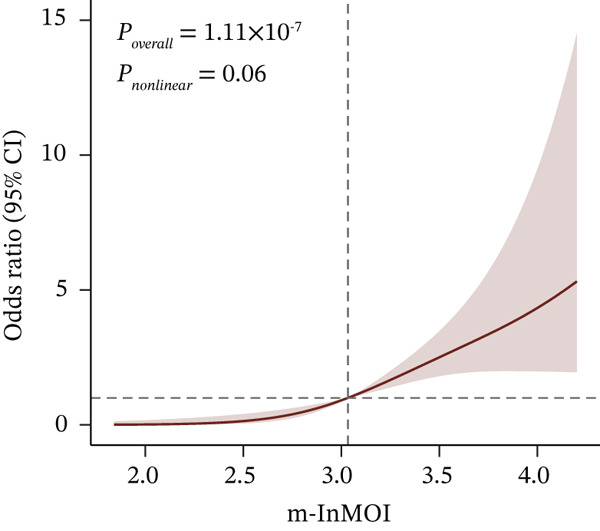
(c)

**Figure:**
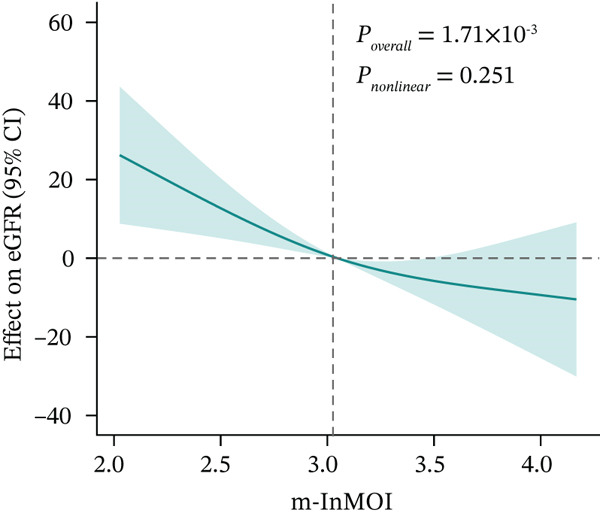
(d)

**Figure:**
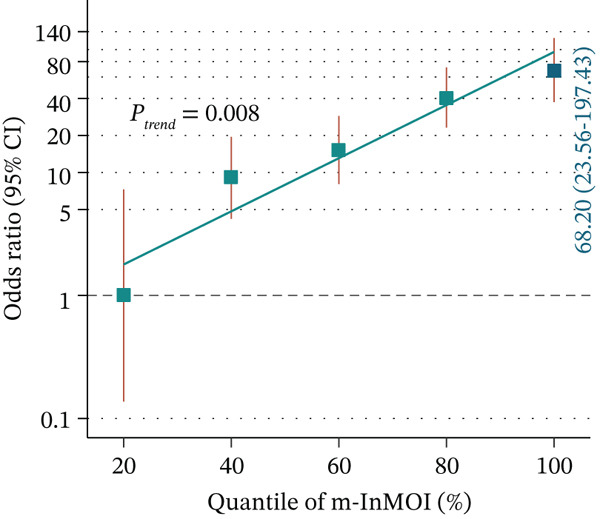
(e)

**Figure:**
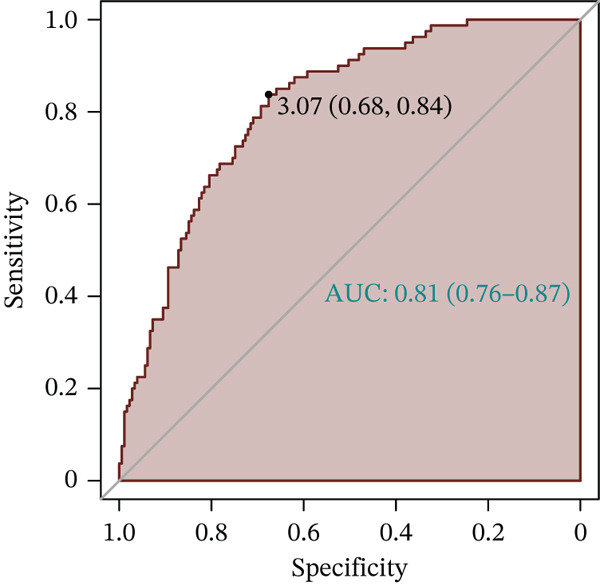
(f)

With the weights substituted, the simplified formula (details in Note S2) is:

m‐InMOI=TyG118×BMI+18.3×VSR

Figure 3b from multivariable regression revealed that the m‐InMOI (OR = 2.01, 95% CI: 1.48~2.74) and history of hypertension (OR = 2.30, 95% CI: 1.32~4.01) were independently associated with renal impairment. Accordingly, the independent significance of m‐InMOI across HTN‐stratified subgroups was assessed (Figure S4), m‐InMOI maintained a significant positive association with renal impairment risk, regardless of HTN status. RCS analysis indicated a linear relationship between the m‐InMOI and the OR for renal impairment, with the 95% CI consistently remaining above unity beyond an m‐InMOI of 3.15 (Figure 3c). Consistent with InMOI, m‐InMOI also displayed a significant linear association with eGFR (Figures 3d and S5), where eGFR declined progressively as m‐InMOI levels increased (P_overall_ < 3.89 × 10^−7^, P_nonlinear_ = 0.37). Quintile analysis showed a significant increasing trend in ORs (*p* for trend < 0.001) (Figure 3e). The screening AUC of the m‐InMOI for renal impairment was 0.81 (95% CI: 0.75~0.87) (Figure 3f).

## 4. Discussion

The present study developed and validated a novel obesity‐metabolism integrated index, the InMOI, for screening renal impairment in early‐stage T2DM. The index combines TyG‐BMI and TyG‐WHR, two composite indicators reflecting metabolic obesity, and demonstrated good discriminative ability with an AUC of 0.78 in the validation population. Furthermore, by incorporating a more precise measure of visceral adiposity, the VSR derived from DEXA, we optimized the index to m‐InMOI, which achieved an improved AUC of 0.81. These results underscore the value of integrating routine metabolic and anthropometric parameters into a practical tool for early identification of renal impairment in young‐onset T2DM patients.

A key finding of our study is the consistent and significant association between TyG‐based composite indices and renal impairment. The TyG index, a reliable surrogate marker of insulin resistance, has previously been linked to diabetic kidney disease [19, 20]. However, insulin resistance often coexists with obesity, which itself contributes to renal dysfunction through multiple pathways such as lipotoxicity, chronic inflammation, and hemodynamic changes [21]. Our results extend previous knowledge by showing that combining the TyG index with indicators of general (BMI) and central obesity (WHR) enhances predictive performance for renal impairment. This aligns with emerging evidence suggesting that fat distribution, particularly visceral fat accumulation, may be more determinative than overall adiposity in the development of diabetic kidney disease. The superior performance of m‐InMOI, which integrates DEXA‐measured VSR, further corroborates the critical role of visceral obesity in the pathogenesis of early renal impairment in T2DM.

Notably, hypertension was identified as an independent indicator of renal impairment in this study, consistent with established literature regarding its role in diabetic nephropathy [22, 23]. Nevertheless, our subgroup analyses further revealed that the diagnosed performance of InMOI remained consistent in both hypertensive and nonhypertensive individuals. These robustness underscores that the indicative value of the InMOI is not merely a byproduct of the confounding effect of hypertension, but rather represents a stable and independent marker of renal health in patients with newly diagnosed T2DM. In contrast, conventional risk factors, such as age, smoking, and alcohol consumption did not exhibit significant associations in this study, a discrepancy that may be attributed to the relatively young age and short diabetes duration of the included patients.

The InMOI and m‐InMOI offer several clinical advantages. First, they are constructed from readily available parameters—WC, HC, TG, and FBG—making them feasible for implementation in routine clinical practice and large‐scale screening programs, especially in resource‐limited settings. Second, the continuous nature of the indices allows for dynamic probability assessment and possible use in tracking patient progress in response to interventions targeting metabolic obesity. The critical point (e.g., InMOI of 2.95 and m‐InMOI of 3.15) may assist in suspicion stratification and guide early referral and intensified management. From a public health perspective, these indices possess significant utility within primary healthcare systems, such as the National Basic Public Health Service Project in China [24]. In these settings, routine physical examinations are widely accessible but often lack standardized or complete laboratory data; specifically, many community health records fail to include renal‐specific markers like the UACR or Scr. InMOI and m‐InMOI can be directly applied to such existing, fragmented physical examination data without the need for large‐scale patient recalls for secondary testing. As a complementary screening tool, this facilitates the rapid identification of individuals who lack formal diagnostic data yet remain at high risk for renal impairment. Such early detection allows for targeted confirmatory testing, definitive clinical intervention, and tailored health education for patients with early‐stage T2DM. Furthermore, for patients who have already undergone DEXA scans for body composition analysis—a common procedure for assessing metabolic health in diabetic populations—clinicians can simultaneously derive high‐precision renal risk assessments via m‐InMOI without additional cost or procedural burden.

Nevertheless, several limitations should be acknowledged when interpreting our findings. First, this study employed specific age and disease‐duration inclusion criteria, which may limit the generalizability of our results to broader populations. By restricting the cohort to adults under 40 with newly diagnosed T2DM, we successfully minimized the confounding effects of age‐related renal degeneration, chronic comorbidities, and long‐term pharmacological interventions. However, this approach inherently reduces the applicability of the InMOI to elderly patients or those with long‐standing diabetes. Second, although we focused on newly diagnosed patients to ensure metabolic homogeneity, we could not fully account for acute fluctuations in TG levels caused by short‐term dietary changes or medications prior to enrollment. Third, as a single‐center study with a limited sample size, our findings may have limited generalizability, and the cross‐sectional design of this study precludes the establishment of causal relationships. Although the InMOI or m‐InMOI showed strong correlation with current renal impairment, prospective studies are warranted to assess its utility in predicting incident diabetic kidney disease and hard renal endpoints over time.

In conclusion, we have developed and validated a simple and effective obesity‐metabolism integrated index for screening renal impairment in early T2DM. The index highlights the synergistic effect of insulin resistance and visceral obesity on renal impairment and provides a practical tool for early identification of high‐risk individuals. Future studies should focus on external validation and evaluating its utility in guiding personalized interventions aimed at preventing diabetic kidney disease.

## Funding

This study was supported by Medical School Joint Innovation and Open Fund of Lishui University (Grant no. X111001).

## Ethics Statement

This retrospective observational study, utilizing real‐world data from the Lishui People′s Hospital Branch of the National Metabolic Management Center (http://national-mmc.com/), was approved by the Hospital′s Ethics Committee (Approval No. 2025‐03‐22), with informed consent obtained from all participants. The ethical approval document is available in the supporting information.

## Conflicts of Interest

The authors declare no conflicts of interest.

## Supporting information


**Supporting Information** Additional supporting information can be found online in the Supporting Information section. Notes S1 and S2 provide detailed derivations for the InMOI and m‐InMOI formulas. Figures S1, S2, S3, S4 and S5 illustrate RCS curves for InMOI and m‐InMOI stratified by hypertension status, variance inflation factors for regression models, TyG‐VSR distribution across groups, and the dose‐response relationship between InMOI and eGFR. Tables S1 and S2 present candidate variable calculation formulas and subgroup analysis results.

## Data Availability

The data that support the findings of this study are available from the corresponding authors upon reasonable request.
