# Supplementary material for: Development and Validation of an Integrated Metabolism‐Obesity Index for Screening Renal Impairment in Patients With Newly Diagnosed Type 2 Diabetes Mellitus
Source: J Diabetes Res. 2026 Apr 13;2026:5164386. doi: 10.1155/jdr/5164386 (PMC13071861; doi:10.1155/jdr/5164386)
Supplement: Supplementary file 1 — Supporting Information Additional supporting information can be found online in the Supporting Information section. Notes S1 and S2 provide detailed derivations for the InMOI and m‐InMOI formulas. Figures S1, S2, S3, S4 and S5 illustrate RCS curves for InMOI and m‐InMOI stratified by hypertension status, variance inflation factors for regression models, TyG‐VSR distribution across groups, and the dose‐response relationship between InMOI and eGFR. Tables S1 and S2 present candidate variable calculation formulas and subgroup analysis results. [file JDR-2026-5164386-s001.docx]

**Supplementary Material**

**Contents**

**Supplementary Notes**

**Supplementary Note 1:** Transparency of InMOI formula derivation

**Supplementary Note 2:** Transparency of m-InMOI formula derivation

**Supplementary Figures**

**Supplementary Figure 1.** RCS curve demonstrating a linear positive association between the InMOI and the OR for renal impairment stratified by hypertension status.

**Supplementary Figure 2.** Variance inflation factors (VIFs) for variables in the regression models.

**Supplementary Figure 3.** Distribution of TyG-VSR levels across renal impairment and non-impairment groups.

**Supplementary Figure 4.** RCS curve demonstrating a linear positive association between the m-InMOI and the OR for renal impairment stratified by hypertension status.

**Supplementary Figure 5.** Dose-response relationship between the InMOI and eGFR.

**Supplementary Tables**

**Supplementary Table 1.** Candidate variables and their calculation formulas.

**Supplementary Table 2.** Subgroup analysis of the association between InMOI or m-InMOI and renal impairment stratified by hypertension status.

**Supplementary Note 1:** Transparency of InMOI formula derivation

**Step 1.** Fundamental formulas:

$InMOI=\beta_{TyG⎼BMI} \times TyG$⎼$BMI+ \beta_{TyG⎼WHR} \times TyG⎼WHR$

Where the interaction terms are defined as:

$TyG$⎼$BMI= TyG \times BMI$

$TyG$⎼$WHR= TyG \times WHR$

**Step 2.** Factoring out the common variable (TyG):

$$InMOI=TyG \times(\beta_{TyG⎼BMI} \times BMI + \beta_{TyG⎼WHR} \times WHR)$$

**Step 3.** Determination of regression coefficients (*β*):

| Variable | Distribution (Mean±SD)^a^ | *β* |
| --- | --- | --- |
| TyG-BMI | 262.41 ± 51.30 | 0.00801 |
| TyG-WHR | 9.25 ± 1.15 | 0.09760 |

^a^~ in the derivation population;

By substituting the *β* values into the model:

$$InMOI=TyG \times(0.00800 \times BMI + 0.00976 \times WHR)$$

The coefficients can be approximated as*:*

$$0.00801= \frac{1}{125}$$

$$0.09760= \frac{12.2}{125}$$

**Step 4.** Factoring out 1/125 to simplify the formula:

$$InMOI= \frac{TyG}{125} \times(BMI + 12.2 \times WHR)$$

**Supplementary Note 2:** Transparency of m-InMOI formula derivation

**Step 1.** Fundamental formulas:

$m⎼InMOI=\beta_{TyG⎼BMI} \times TyG$⎼$BMI+ \beta_{TyG⎼VSR} \times TyG⎼VSR$

Where the interaction terms are defined as:

$TyG$⎼$BMI= TyG \times BMI$

$TyG$⎼$VSR= TyG \times VSR$

**Step 2.** Factoring out the common variable (TyG):

$$m⎼InMOI=TyG \times(\beta_{TyG⎼BMI} \times BMI + \beta_{TyG⎼VSR} \times VSR)$$

**Step 3.** Determination of regression coefficients (*β*):

| Variable | Distribution (Mean ± SD)^a^ | *β* |
| --- | --- | --- |
| TyG-BMI | 262.41 ± 51.30 | 0.00847 |
| TyG-VSR | 5.17 ± 1.90 | 0.15508 |

^a^~ in the derivation population;

By substituting the *β* values into the model:

$$m⎼InMOI=TyG \times(0.00847 \times BMI + 0.15508 \times VSR)$$

The coefficients can be approximated as*:*

$$0.00847= \frac{1}{118}$$

$$0.15508= \frac{18.3}{118}$$

**Step 4.** Factoring out 1/118 to simplify the formula:

$$m⎼InMOI= \frac{TyG}{118} \times(BMI + 18.3 \times VSR)$$

**Supplementary Figure 1.** RCS curve demonstrating a linear positive association between the InMOI and the OR for renal impairment stratified by hypertension status.


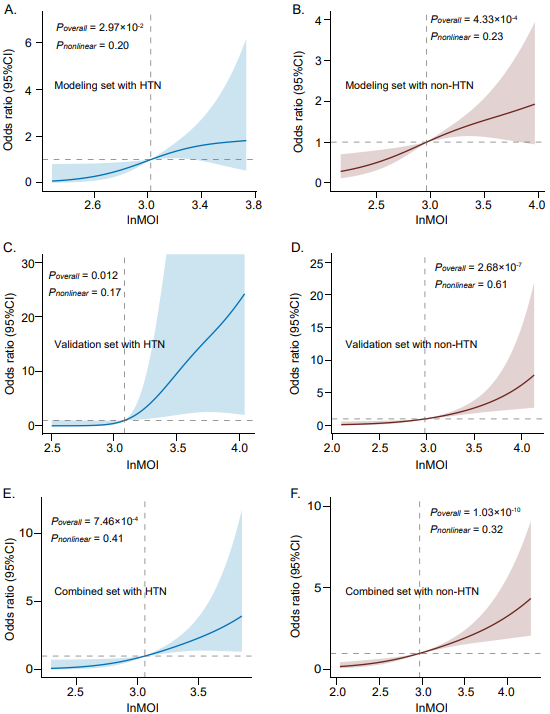


Stratified analysis of InMOI and renal impairment risk in the modeling cohort (A: with HTN; B: without HTN), validation cohort (C: with HTN; D: without HTN), and combined cohort (E: with HTN; F: without HTN). Adjusted for age, sex, smoking, alcohol consumption. Abbreviation: HTN, presence of hypertension; non-HTN, absence of hypertension.

**Supplementary Figure 2.** Variance inflation factors (VIFs) for variables in the regression models.


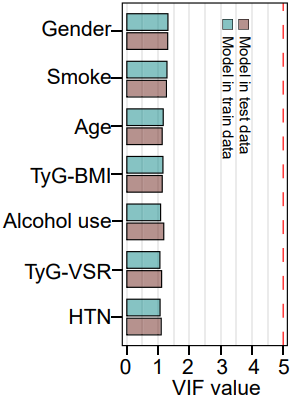


The bar plot illustrates that all VIF values remained below 5, indicating no significant multicollinearity between TyG-related indices and other covariates in both cohorts**.**

**Supplementary Figure 3.** Distribution of TyG-VSR levels across renal impairment and non-impairment groups.


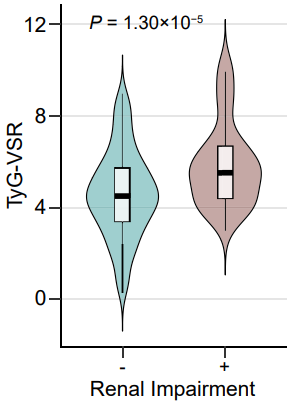


The violin plot illustrates that TyG-VSR levels were significantly higher in T2DM patients with renal impairment compared to those without impairment in the validation cohort.

**Supplementary Figure 4.** RCS curve demonstrating a linear positive association between the m-InMOI and the OR for renal impairment stratified by hypertension status.


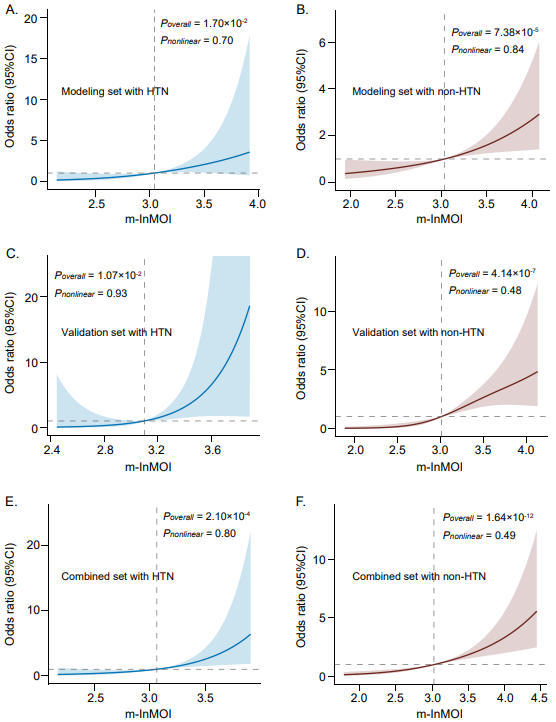


Stratified analysis of InMOI and renal impairment risk in the modeling cohort (A: with HTN; B: without HTN), validation cohort (C: with HTN; D: without HTN), and combined cohort (E: with HTN; F: without HTN). Adjusted for age, sex, smoking, alcohol consumption. Abbreviation: HTN, presence of hypertension; non-HTN, absence of hypertension.

**Supplementary Figure 5.** Dose-response relationship between the InMOI and eGFR.


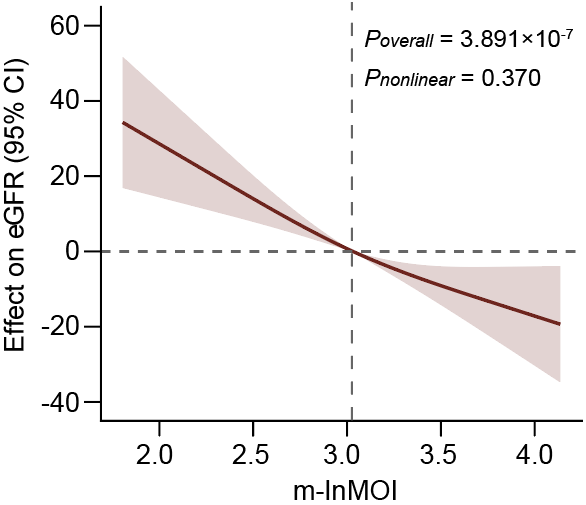


The RCS curve illustrates a significant non-linear positive association between the InMOI and eGFR levels (*P_overall_* < 3.891×10^-7^, *P_nonlinear_* = 0.370). The risk of elevated eGFR increases more steeply beyond a certain InMOI threshold.

**Supplementary Table 1.** Candidate variables and their calculation formulas.

| Indicators | Formulas |
| --- | --- |
| Body Mass Index (BMI) | $\frac{Weight (kg)}{{Heigth (m)}^{2}}$ |
| Body Roundness Index (BRI) | $364.2-365.5 \times\sqrt{1-{(\frac{WC (m)}{2\pi\times(0.5 \times Heigth (m))})}^{2}}$ |
| Geriatric Nutritional Risk Index (GNRI) | $1.489 \times ALB \left( g/L \right)+41.7 \times\frac{Weight (kg)}{22 \times{Heigth (m)}^{2}}$ |
| Weight-Adjusted-Waist Index (WWI) | $\frac{WC (cm)}{\sqrt{Weight (kg)}}$ |
| A Body Shape Index (ABSI) | $\frac{WC (m)}{\sqrt[3]{{BMI}^{2}} \times\sqrt{Height (m)}}$ |
| Waist-to-Height Ratio (WHtR) | $\frac{WC (m)}{Height (m)}$ |
| Waist-to-Hip Ratio (WHR) | $\frac{WC (m)}{HC (m)}$ |
| Visceral-to-Subcutaneous fat area Ratio (VSR) | $\frac{VFA\left( {cm}^{2} \right)}{SFA({cm}^{2})}$ |
| Relative Fat Mass (RFM) | $64-\left( 20 \times\frac{Height \left( m \right)}{WC\left( m \right)} \right)+(12 \times Gender)$ |
| estimated Glomerular Filtration Rate (eGFR) | $186 \times{Scr (mg/dL)}^{-1.154} \times{Age}^{-0.203} \times Gender$ |
| Triglyceride-Glucose Index (TyG) | $\frac{ln[TG (mg/dL) \times FBG (mg/dL)]}{2}$ |
| TyG-Waist Circumference (TyG-WC) | $TyG \times WC$ |
| TyG-BMI | $TyG \times BMI$ |
| TyG-BRI | $TyG \times BRI$ |
| TyG-GNRI | $TyG \times GNRI$ |
| TyG-WWI | $TyG \times WWI$ |
| TyG-ABSI | $TyG \times ABSI$ |
| TyG-WHtR | $TyG \times WHtR$ |
| TyG-WHR | $TyG \times WHR$ |
| TyG-RFM | $TyG \times RFM$ |
| TyG-VSR | $TyG \times VSR$ |

Abbreviations in formulas: WC, waist circumference; ALB, serum albumin; HC, hip circumference; Scr, serum creatinine; TG, triglycerides; FBG, fasting plasma glucose.

**Supplementary Table 2.** Subgroup analysis of the association between InMOI or m-InMOI and renal impairment stratified by hypertension status.

| **Data Set** | **HTN** | **No.** | **InMOI** | | |  | **m-InMOI** | | |
| --- | --- | --- | --- | --- | --- | --- | --- | --- | --- |
|  |  |  | **OR** | **95%CI** | ***P*** |  | **OR** | **95%CI** | ***P*** |
| Modeling | Yes | 73 | 5.45 | 1.68-21.41 | 7.95×10^-3^ |  | 5.72 | 1.92-20.97 | 3.78×10^-3^ |
|  | No | 314 | 2.65 | 1.63-4.41 | 1.13×10^-4^ |  | 2.65 | 1.73-4.16 | 1.38×10^-5^ |
| Validation | Yes | 47 | 72.4 | 6.58-2378.02 | 3.49×10^-3^ |  | 44.17 | 5.54-825.51 | 2.44×10^-3^ |
|  | No | 212 | 6.92 | 3.62-14.36 | 3.29×10^-8^ |  | 7.80 | 4.13-16.13 | 3.02×10^-9^ |
| Combined | Yes | 120 | 8.38 | 3.05-27.14 | 1.20×10^-4^ |  | 7.81 | 3.11-22.82 | 4.58×10^-5^ |
|  | No | 526 | 3.84 | 2.63-5.73 | 1.18×10^-11^ |  | 3.91 | 2.76-5.67 | 1.08×10^-13^ |

Adjusted for age, sex, smoking, alcohol consumption
